# Supplementary material for: Del1 Is a Growth Factor for Skeletal Progenitor Cells in the Fracture Callus
Source: Biomolecules. 2023 Aug 3;13(8):1214. doi: 10.3390/biom13081214 (PMC10452420; doi:10.3390/biom13081214)
Supplement: Supplementary file 1 [file biomolecules-13-01214-s001.zip › Supplemental Figure S1.pdf]

Supplement Figure S1. Immunohistochemical staining for non-skeletal lineages.

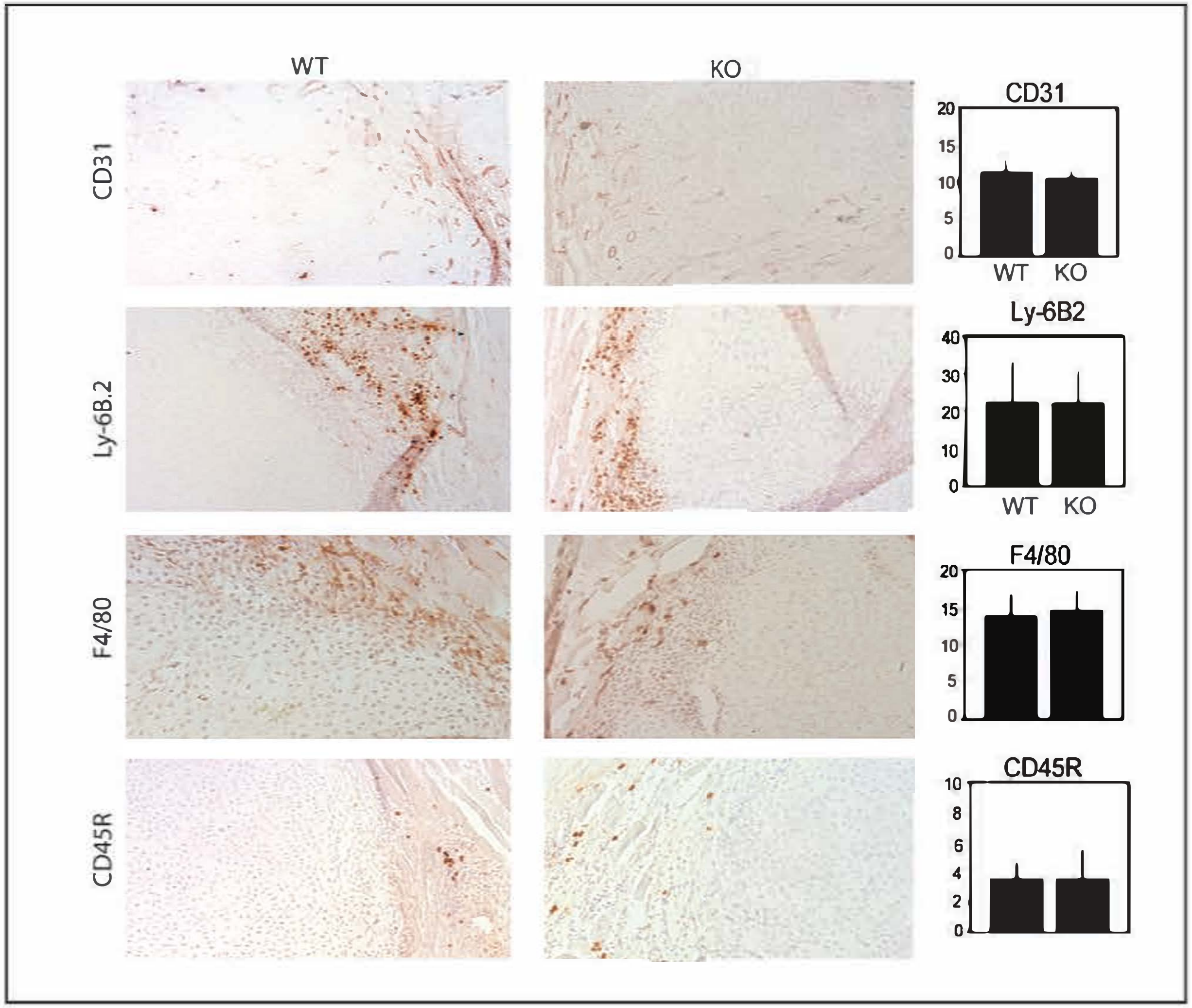

Immunohistochemical staining for non-skeletal lineages. Histology analysis of the bone fracture callus for endothelial cells (CD31) macrophage (F4/80), neutrophil and monocytes (Ly-6B.2), and T call and B cells (CD45).
