# Supplementary material for: Del1 Is a Growth Factor for Skeletal Progenitor Cells in the Fracture Callus
Source: Biomolecules. 2023 Aug 3;13(8):1214. doi: 10.3390/biom13081214 (PMC10452420; doi:10.3390/biom13081214)
Supplement: Supplementary file 1 [file biomolecules-13-01214-s001.zip › Supplemental Figure S2.pdf]

Supplement Figure S2. Exogeneous Del1 protein elution from hydrogel.

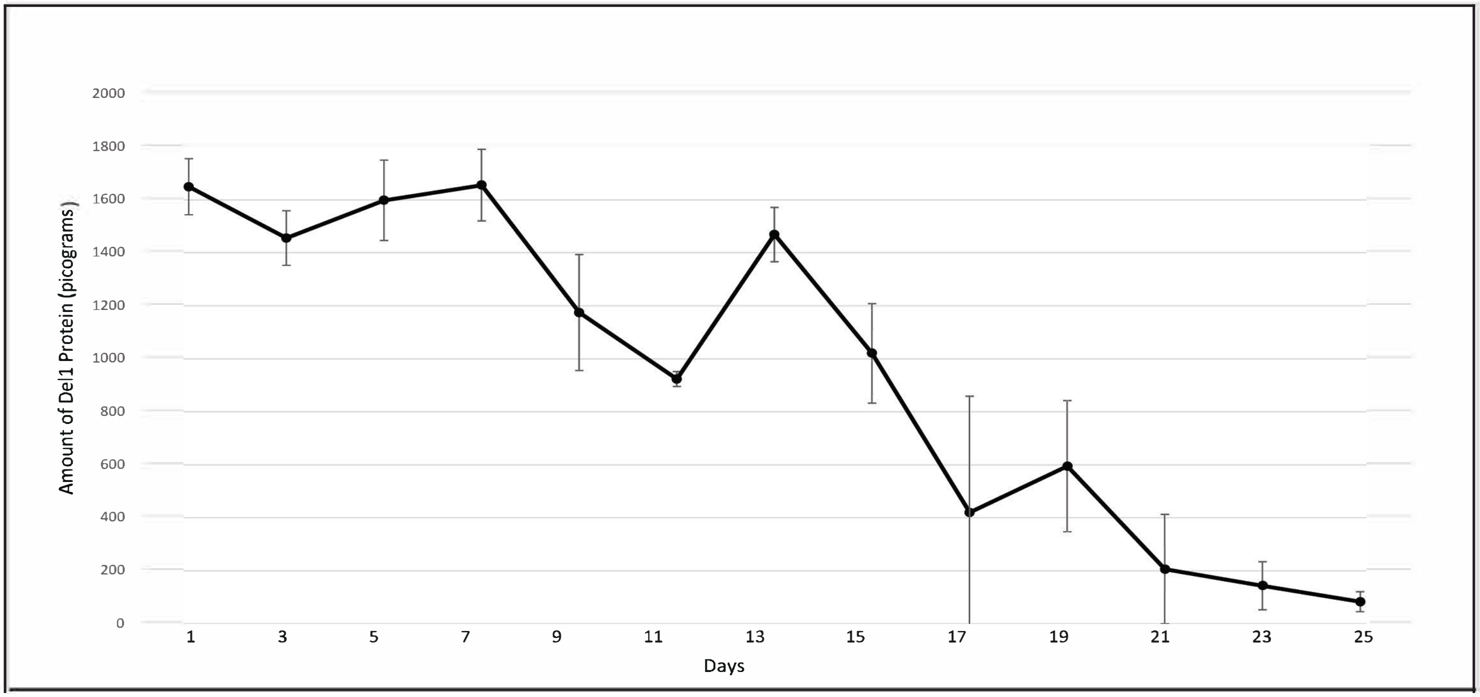

Exogeneous Del1 elution from hydrogel. Hydrogel with Del1 was placed in standard petri dish and the amount of Del1 elution was measured. The results showed consistent release of Del1 from hydrogel for 28 days. (n=3)
